# Return to sport after acetabular and pelvic ring fractures in amateur athletes: A retrospective study

**DOI:** 10.1186/s10195-025-00876-5

**Published:** 2025-10-31

**Authors:** Giuseppe Rovere, Amarildo Smakaj, Domenico De Mauro, Vincenzo Mattiacci, Giovanni Vicenti, Francesco Bosco, Lawrence Camarda, Pasquale Farsetti, Francesco Liuzza

**Affiliations:** 1 Department of Orthopaedics and Traumatology, Policlinico Tor Vergata Foundation, Rome, Italy; 2 Department of Clinical Sciences and Translational Medicine, Tor Vergata University of Rome, Rome, Italy; 3 Department of Biomedicine and Prevention, Tor Vergata University of Rome, Rome, Italy; 4 Department of Orthopedics, Ageing and Rheumatological Sciences, Fondazione Policlinico Universitario Agostino Gemelli IRCCS, 00168 Rome, Italy; 5 Department of Public Health, Orthopedic Unit, Federico II University, Via Sergio Pansini, 5, 80131 Naples, Italy; 6 Department of Orthopaedics and Traumatology, University of Turin, Turin, Italy; 7 Department of Basic Medical Sciences, Neuroscience and Sense Organs, School of Medicine, Aldo Moro University, 70121 Bari, Italy; 8 Orthopedics and Traumatology Unit, Azienda Ospedaliero Universitaria Consorziale Policlinico Giovanni XXIII, 70124 Bari, Italy; 9 Department of Orthopaedics and Traumatology, University of Palermo, 90133 Palermo, Italy

**Keywords:** Acetabular fractures, Pelvic ring fractures, Return to sport, Functional recovery, Amateur athletes

## Abstract

**Background:**

Acetabular fractures are complex hip injuries with high social and economic costs, as they affect individuals of working age. These fractures often result in long-term complications, including chronic pain and sexual dysfunction, which impair quality of life and limit physical activity. With growing interest in fitness, understanding factors that impact return to sport post injury is critical. This study examines how fracture type and surgical approach influence functional recovery and return to sport after acetabular and pelvic fractures.

**Material and methods:**

This retrospective cohort study analyzed outcomes in patients with acetabular and pelvic ring fractures, focusing on their ability to return to sport. Patients treated between 2018 and 2022 at Policlinico Universitario A. Gemelli in Rome were included, specifically those with isolated fractures managed by open reduction internal fixation (ORIF) or closed reduction internal fixation (CRIF) techniques. Demographic and clinical data were collected, and fractures were categorized by Judet–Letournel (acetabular) or Young–Burgess (pelvic ring) classifications. Outcomes were assessed using four physical activity-related scores: Hip Sport Activity Scale (HSAS), Hip Outcome Score (HOS), Tegner Activity Scale (TAS), and Modifiable Activity Questionnaire (MAQ), alongside the 12-Item Short Form Health Survey (SF-12) for quality of life. Eligible participants, aged 18–65 years, had no prior surgeries or neurological/cardiopulmonary diseases, nor concurrent limb fractures or severe trauma. Data analysis used Student’s *t*-test and chi-squared tests for continuous and categorical variables, respectively, with analysis of variance (ANOVA) for subgroup comparisons.

**Results:**

The study included 35 patients, with 20 acetabular fractures (4:1 male/female ratio, average age 45.3 years) and 15 pelvic ring fractures (6.5:1 male/female ratio, average age 51.3 years). Follow-up averaged 1074 days for the acetabular group and 1446 days for the pelvic group. Clinical outcomes showed that both groups had similar Physical Component Summary (PCS-12) and Mental Component Summary (MCS-12) scores, with no statistically significant differences (*p* > 0.05). Total MAQ scores were higher in the pelvic group, mainly owing to higher work-related activity scores, while sport-related scores were similar. Hip Outcome Scores (HOS) also indicated comparable function in daily activities and sports, with acetabular fractures scoring 72.2 and pelvic fractures scoring 74.8. HSAS and TAS measures showed no significant difference between groups. Subgroup analysis found no significant outcome differences on the basis of fracture location (anterior versus posterior acetabulum or pelvic ring patterns).

**Conclusions:**

The study found no statistically significant differences in return-to-sport outcomes between acetabular and pelvic ring fractures, highlighting the complexity of both injuries. Future research with larger samples and standardized functional scores is recommended for clearer insights into recovery outcomes.

**Level of evidence:**

III

## Introduction

Acetabular fractures are challenging intra-articular hip lesions that represent an emerging problem in traumatology. Although in recent decades the average age is increasing, epidemiological studies show that the mean age of pelvic fractures has risen from 38.6 to 45.2 years [1, 2]. Morbidity and social costs remain high because these patients are still of working age and require demanding outcomes [3]. In addition to the acute morbidity and mortality associated with pelvic fractures, long-term complications such as chronic pain, sexual dysfunction, and post-traumatic stress disorder can significantly impact the quality of life for these patients [4, 5]. It is crucial to predict patients’ outcomes, including return to sport, and inform them about the potential challenges and milestones post injury. Sexual dysfunction is one of the most extensively studied aspects of this type of injury. Notably, it has been found that the occurrence of sexual dysfunction varies significantly on the basis of the specific pattern of sustained fracture [6, 7]. The growing interest in physical activity and fitness highlights the importance of considering the impact of pelvic fractures on individuals’ ability to return to amateur sports after trauma [8]. Returning to sport after musculoskeletal injuries represents a significant milestone for individuals on their road to recovery [9]. However, post-traumatic hip osteoarthritis is a known complication after pelvic fractures that can greatly affect an individual’s ability to engage in sports and physical activities. Many other complications following pelvic fractures cause impaired hip function such as avascular necrosis of the femoral head, nerve damage, and heterotopic ossifications [10]. These complications can significantly impact an individual’s ability to participate in sporting activities and may also lead to long-term disability. Even if return to sport following other types of fractures and musculoskeletal injuries has been extensively studied [11, 12], there is limited research available on returning to sporting activities after pelvic fractures [13].

The study aims to compare patient outcomes for acetabular and pelvic ring fractures in relation to returning to sports. This study aims to assess how the location and type of fracture, as well as the surgical approach used, affect functional recovery and return-to-sport activities.

## Materials and methods

This is a retrospective cohort study that compared patients affected by acetabular fracture with those with pelvic fracture in relation to returning to sports. The study analyzed different fracture patterns in both acetabular and pelvic ring fracture, all treated with open reduction internal fixation (ORIF) or closed reduction internal fixation (CRIF) techniques. Patients who were treated at Policlinico Universitario A. Gemelli in Rome between 2018 and 2022 were included in the study. This retrospective study was conducted using anonymized data.

Formal approval by an ethics committee was not required and was therefore waived. Patients were identified through a manual review of departmental surgical registries and discharge summaries on the basis of procedural descriptions of acetabular and pelvic ring fracture fixation. Standardized International Classification of Diseases (ICD) codes were not used, as they are not consistently implemented for surgical case retrieval in our institution.

Hospital records and clinical notes were reviewed to collect demographic data, injury mechanism, date of surgery, operating times, type of implants, and surgical approaches. Mechanisms of injury were also recorded and included road traffic accidents, falls from height, and in a minority of cases, sports-related trauma such as cycling incidents. Acetabular fractures were classified according to Judet–Letournel classification, whereas pelvic ring fractures were classified according to Young–Burgess classification.

Clinical outcomes were evaluated with patient-reported outcomes (PROMs). As there are not questionnaires that specifically assess return-to-sport activity after acetabular and pelvic ring fractures, four scores that assess physical activity were used. Among these, Hip Sport Activity Scale (HSAS) and Hip Outcome Score (HOS) are specifically designed to evaluate physically active patients with hip disease but without severe degenerative change. Tegner Activity Scale (TAS) and Modifiable Activity Questionnaire (MAQ) are self-reported questionnaires that assess general prior and post-injury level of activity. The 12-Item Short-Form Health Survey (SF-12) was also administrated to assess health-related quality of life.

To be eligible for the study, participants should have sustained isolated acetabular or pelvic fracture. Potential patients with concomitant limb fractures were excluded from the study, as their presence could potentially impact clinical outcomes and introduce confounding biases. Similarly, patients with severe head trauma requiring surgery or severe abdominal or thoracic trauma were also excluded for the same reasons. Moreover, patients had to have been in the age range of 18–65 years to be enrolled in the study. Individuals with no history of previous sports activity were not considered for participation in the study. Information on prior sports activity was obtained from clinical records, where functional history, including engagement in regular amateur sports, was routinely documented during the initial clinical evaluation or preoperative assessment. Prospective participants had to commit to a follow-up period of at least 1 year to be included in the study. Individuals who underwent hip or pelvic surgeries in the past were excluded from participation. Individuals with a history of neurologic and cardiopulmonary diseases were not eligible for inclusion in the study. Patients presenting with concomitant acetabular and pelvic ring fractures were excluded from the study, as these combined injuries were considered a potential source of bias owing to their greater complexity and impact on functional outcomes.

Data are reported in terms of the mean and standard deviation for continuous variables, while categorical variables are presented as frequency distributions (in percentages). Missing data were acknowledged and retained in the analysis. To reduce potential sources of bias, strict adherence to the predefined inclusion criteria was maintained, co-interventions were avoided, data completeness was prioritized, and outcomes were reported according to predefined criteria. Statistical analysis was performed using the Student’s *t*-test for continuous variables and the chi-squared test for categorical variables. Subgroup functional outcomes were compared using a one-factor analysis of variance (ANOVA) test. A *p*-value of < 0.05 was considered the threshold for statistical significance in all analyses.

## Results

A total of 57 patients were initially screened for eligibility. However, 29 were excluded owing to the following criteria: concomitant lower limb fractures (*n* = 11); polytrauma involving severe abdominal, thoracic, or head injuries (*n* = 8); previous pelvic or hip surgery (*n* = 3); age outside the 18–65-year range (*n* = 2); and follow-up shorter than 12 months (*n* = 5). The research involved 28 participants in total. Among them, 15 individuals had acetabular fractures, with a male/female ratio of 4:1 and an average age of 45.3 ± 13.79 years. In contrast, the number of patients with pelvic ring fractures was 13, featuring a male/female ratio of 6.5:1 and a slightly higher mean age of 51.3 ± 8.97 years. The mean follow-up in the acetabular group was 1074.22 ± 402.31 days, compared with 1446.73 ± 310.60 days in the pelvic ring group (Table 1). The mean time from injury to surgery was 2.3 ± 1.2 days. The number of acetabular and pelvic fracture patterns is described in Table 2 according to the respective chosen classifications. Road traffic accidents accounted for the majority of injuries in both groups, followed by falls from height. Sports-related injuries were infrequent.

**Table 1:** Demographic data

	Acetabulum	Pelvic ring
Number of patients	15	13
Male/female ratio	4:1	6.5:1
Age, years	45.3 ± 13.79	51.3 ± 8.97
Follow-up (days)	1074.22 ± 402.31	1446.73 ± 310.60

**Table 2:** Acetabular^*^ and pelvic ring^**^ fracture classification

Acetabular fractures		Pelvic ring fracture	
Posterior wall	4	Vertical shear	2
Posterior column and wall	1	APC I	2
Anterior wall	4	APC II	3
Anterior column	1	APC III	1
Posterior wall and column	1	LC I	2
Anterior column and wall	1	LC II	3
Transverse with Posterior wall	1	LC III	0
Both columns	2		
Total	15		13

^*^According to Judet–Letournel

^**^According to Young–Burgess

Patients with acetabular fractures had a PCS-12 of 45.81 (± 2.77) and an MCS-12 of 42.78 (± 2.50), while patients with pelvic fracture had a PCS-12 of 45.36 (± 2.73) and an MCS-12 of 47.32 (± 3.00).

In acetabular fractures, the total MAQ score was 64.79 ± 15.54, with sport-related items at 35.26 ± 8.166 and work-related items at 29.53 ± 11.43. For pelvic fractures, the total MAQ score was 131.8 ± 36.93, with sport-related items at 39.61 ± 11.82 and work-related items at 92.17 ± 34.70. The statistical analysis revealed a *p*-value of 0.056 when comparing acetabular and pelvic ring outcomes, therefore the difference was not considered statistically significant.

The total HOS score for acetabular fractures was 72.22 ± 5.32, with the activities of daily living (ADL)-related item at 75.25 ± 4.88 and the sport-related outcome at 66.06 ± 5.57. Pelvic fractures resulted in a HOS score of 74.80 ± 5.44, with the ADL component measuring 80.93 ± 4.86 and sport-related outcomes measuring 65.14 ± 7.89. The statistical analysis found a *p*-value > 0.05 when comparing acetabular and pelvic ring outcomes, indicating that the difference was not considered to be statistically significant.

Acetabular fractures had an average HSAS of 3.50 ± 0.48, while pelvic fractures had an average HSAS of 2.47 ± 0.44. The difference in HSAS between acetabular and pelvic fractures was not found to be significant.

Acetabular fractures had an average TAS of 3.75 ± 0.49, while pelvic fractures had an average TAS of 3.40 ± 0.48. There was no significant difference in TAS between acetabular and pelvic fractures. Scores for specific acetabular and pelvic fracture patterns are presented in Tables 3 and 4, respectively.

**Table 3:** Acetabular fracture outcomes

	SF-12		MAQ			HOS			HSAS	TAS
	PCS-12	MCS-12	Sport	Work	Total	ADL	Sport	Total		
Posterior wall	60.8	33.6	0.00	0.00	0.00	97%	78%	90%	5	7
	30.5	38.8	0.00	85.10	85.10	35%	30%	33%	1	1
	45.9	42.3	9.22	147.70	156.92	76%	83%	89%	2	3
	30.3	58.3	24.40	0.00	24.40	82%			1	3
	41.875	43.25	8.405	58.2	66.605	72.50%	63.67%	70.67%	2.25	3.5
Anterior column	35.6	26.3	5.52	0.00	5.52	66%	25%	52%	1	0
	60.7	28.4	3.4	0.00	3.40	75%			2	3
	58.8	30	6.78	0.00	6.78	90%	97%	92%	4	3
	45	52.9	24.7	0.00	24.70	78%	58%	71%	3	3
	50.025	34.4	10.1	0	10.1	77.25%	60.00%	71.67%	2.5	2.25
Posterior column and posterior wall	46.4	39	26.2	0.00	26.20	75%	50%	66%	2	3
Both columns	56.6	60.7	61.7	110.70	172.40	100%	100%	100%	6	7
	30.8	52.8	16.6	0.00	16.60	25%	42%	31%	2	3
	43.7	56.75	39.15	55.35	94.5	0.625	0.71	0.655	4	5
Transverse and posterior wall	26.2	33.7	26.20	0.00	26.20	38%	36%	35%	1	1
Posterior column and anterior wall	28.1	46.7	49.26	114.22	163.48	63%	53%	60%	3	3
Anterior wall and columns	57.8	46.8	116.8	0.00	116.80	93%	89%	91%	4	3

**Table 4:** Pelvic ring fracture outcomes

	SF-12		MAQ			HOS			HSAS	TAS
	PCS-12	MCS-12	Sport	Work	Total	ADL	Sport	Total		
VS	27.50	41.10	5.53	0.00	5.53	55.00	22.00	44.00	2	1
	38.00	47.10	128.20	110.80	239	64.00	64.00	64.00	2	3
	32.75	44.10	66.87	55.40	122.27	59.50	43.00	54.00	2.00	2.00
APC	55.60	60.70	15.48	0.00	15.48	94.00	78.00	88.00	3	4
	26.80	37.80	0.00	0.00	0.00	35.00	5.00	25.00	2	1
	56.60%	29.30%	40.70	0.00	40.70	92%	91%	92%	1	4
	54.80	57.00	20.80	173.00	193.8	100.00	100.00	100.00	5	4
	43.50	58.60	0.00	0.00	0.00	88.00	86.00	87.00	4	5
	52.10	56.80	0.00	0.00	0.00	78.00	55.00	70.00	1	2
	38.89	45.20	12.83	28.83	41.66	65.99	54.15	61.82	2.67	3.33
LC	30.80	51.30	0.00	0.00	0.00	65.00	31.00	63.00	0	3
	47.80	53.80	20.80	299.00	319.8	91.00	0.00	63.00	0	5
	41.00	50.40	20.50	0.00	20.5	74.00	62.00	69.00	2	1
	47.00	32.00	0.00	0.00	0.00	93.00	66.00	84.00	5	4
	59.60	24.20	55.40	99.70	155.1	97.00	97.00	97.00	2	3
	45.24	42.34	19.34	79.74	99.08	84.00	51.20	75.20	1.80	3.20

*VS* vertical shear, *APC* anterior–posterior compression, *LC* lateral compression

Fractures involving the posterior components of the acetabulum showed better clinical outcomes in terms of MAQ (74.19 ± 23.71 versus 30.57 ± 17.72), TAS (3.44 ± 0.73 versus 2.40 ± 0.60), and MCS-12 (45.10 ± 3.39 versus 36.88 ± 5.41). However, a subgroup analysis of acetabulum fracture patterns showed no discernible variations in any of the evaluated scores, regardless of the anterior or posterior component involvement. Similarly, there were no statistically significant differences in the investigated outcome scores for vertical shear (VS), anterior–posterior compression (APC), and lateral compression (LC) fractures of the pelvic ring.

## Discussion

Acetabular and pelvic ring fractures are still some of the most challenging fractures in terms of outcome, especially when it comes to returning to sports [14]. This study does not intend to suggest clinical equivalence between acetabular and pelvic ring fractures. Rather, it seeks to explore functional outcomes in terms of return to sport among patients undergoing surgical management for two distinct, yet regionally related, fracture types. This comparison is observational in nature and not inferential. They are often associated with a high rate of complications, including post-traumatic arthritis, and the high-energy trauma frequently involves other vital systems, making treatment particularly delicate [15].

As surgical skills continue to advance and knowledge increases, it has become increasingly common to find patients achieving a good functional outcome and expressing a desire to return to their normal lifestyle.

Having said that, it is important to explore and understand the capacity of return to sport in those fractures. When looking at the data, a clear difference could not be demonstrated between acetabular and pelvic ring fractures on the basis of some questionnaires (SF-12, HOS), which suggest that pelvic ring fractures have a better outcome than acetabular fractures, while others indicate the opposite. However, these findings are not statistically significant. The MAQ score in acetabulum and pelvic ring fractures is the only score that showed a statistical value close to significance, which could become more pronounced with an increase in the sample size of patients. In our review of acetabular fractures, we found that anterior fractures generally result in slightly better outcomes for returning to sport compared with those in the posterior region. This could be attributed to the need for different surgical approaches for each type of fracture. In posterior-region fractures, it is common practice to dissect the femur’s extrarotators, which can harm the muscular compartment and reduce functional ability. In addition, there is a higher rate of post-traumatic arthritis, which contributes to worse outcomes. Although most injuries were unrelated to sports, the role of trauma dynamics—particularly in cycling or high-impact athletic accidents—should be considered when interpreting functional outcomes.

Anterior–posterior compression fractures appeared to be associated with better outcomes compared with lateral compression and vertical shear patterns among pelvic ring injuries. This difference may be attributed to the distinct lesion patterns: APC is characterized by pubic diastasis and, in severe cases, disruption-diastasis of both anterior and posterior sacroiliac joint ligaments with dislocation. LC fractures, while the most common type, are associated with rotational and vertical instability. The worst results were observed in VS lesions, which are the most severe and unstable type often accompanied by visceral injuries.

To this day, literature on the ability to return to sport is very limited [13, 16]. For example, Monteleone et al. (2023) analyzed pelvic ring fractures separately, emphasizing the importance of studying these injuries individually. Their approach highlights the existing fragmentation in the literature, and the need for more standardized and stratified analyses, especially regarding return to sport. However, much of the research focuses on returning to work and sexual dysfunction [4, 17, 18]. The majority of the available information about return to sport comes from studies that examined young athletes with pelvic avulsion fractures [19, 20]. However, these studies may not be directly relevant to the subject of this research.

One of the first sets of data about the return to sport after acetabular and pelvic fracture comes from Kheir et al. [21]. They reported that following surgery, most patients were able to resume their sports activities. The poorest outlook is associated with fractures involving both columns and the posterior wall of the acetabulum. Middle-aged patients showed better recovery compared with both younger and elderly patients in each group [21].

The impact of operatively treated pelvic fractures on sporting and physical activities, as well as risk factors for decreased activity, was further investigated in 2014 by Harvey-Kel et al. [13], without distinguishing between acetabular and pelvic ring fractures. They found predictive links between pre-injury sporting activity levels and post-injury activity levels, with a decrease in sporting activity correlating with lower quality of life, concluding that patients should be informed about potential limitations on sporting activities following surgical treatment for a pelvic fracture [13]. Although acetabular and pelvic ring fractures differ substantially in biomechanics, injury mechanisms, and surgical treatment, they both involve the pelvic region and may equally impact physical function and return to sport. In real-world orthopedic practice, these injuries are often encountered in overlapping patient populations. Therefore, understanding and comparing their functional consequences—particularly in amateur athletes—can provide useful clinical insights. This exploratory comparison aims to identify whether different fracture patterns lead to divergent or convergent recovery profiles, acknowledging the biological and anatomical differences between them. One limitation of these studies is the absence of a reproducible score for return to sport. Instead, the study relies on a specifically designed questionnaire that has not yet been validate. The literature review shows that there is still no validated score for return-to-sports activity in these patients. A comprehensive investigation on return to work, sport activity, and sexual dysfunctions was performed by Monteleone et al. [16], exclusively on pelvic ring fractures. They used HHS and TAS to evaluate sporting outcomes, which aligns with our findings [16].

Although variations in functional outcomes were observed among different fracture types, none reached statistical significance. It is important to acknowledge that certain study limitations may have influenced these results. The small sample size and retrospective nature of the study, along with the presence of different first operators, may have affected therapeutic success depending on surgical skills. Owing to the limited number of patients in each fracture subtype, functional outcome scores were not stratified according to the detailed Young–Burgess classification, as such subgroup analysis was deemed underpowered and unlikely to yield meaningful clinical insights. A larger sample size and a prospective design could help mitigate these limitations in future research efforts.

One significant limitation that emerged from our study is the variation in results across different scores due to their lack of specificity. This limitation has also been noted in other studies, suggesting a need for a functional score that is more specific to enhance result consistency. Finally, the absence of statistically significant differences between acetabular and pelvic ring fractures may reflect methodological limitations rather than actual equivalence. The small sample size, retrospective design, and inclusion of heterogeneous fracture patterns may have reduced the power to detect meaningful differences between groups.

## Conclusions

The comparison between acetabular and pelvic ring fractures revealed some interesting findings and insights in terms of an emerging clinical outcome—the return to sport. However, despite the observed variations, the lack of statistically significant differences underscores the complexity and challenges associated with both acetabular and pelvic ring fractures. Moving forward, future research should aim to address these limitations by incorporating larger sample sizes and prospective study designs to obtain more conclusive and reliable findings. In addition, the development of more specific and standardized functional scores about returning to sport could enhance result consistency and provide deeper insights into the functional outcomes of these challenging fractures.

## Data Availability

The datasets used and/or analyzed during the current study are available from the corresponding author on reasonable request.
